# Association between Common Allergic Symptoms and Cancer in the NHANES III Female Cohort

**DOI:** 10.1371/journal.pone.0042896

**Published:** 2012-09-18

**Authors:** Young Kwang Chae, Stefan Neagu, Jongoh Kim, Athanasios Smyrlis, Mahasweta Gooptu, William Tester

**Affiliations:** 1 Division of Cancer Medicine, University of Texas MD Anderson Cancer Center, Houston, Texas, United States of America; 2 Department of Medicine, Christiana Healthcare System, Newark, Delaware, United States of America; 3 Department of Medicine, Baylor College of Medicine, Houston, Texas, United States of America; 4 Department of Medicine, Albert Einstein Medical Center, Philadelphia, Pennsylvania, United States of America; UCLA, United States of America

## Abstract

**Background:**

Previous epidemiological studies have investigated the association between allergic symptoms and cancer occurrence. However, the role of allergy in cancer has been elusive, especially for the female population.

**Methods:**

We examined the relationship between cancer prevalence and common allergic symptoms of rhinoconjunctivitis (RC) and wheezing (WZ) among NHANES III female participants.

**Results:**

Among 4600 people, 36.3% (n = 1669) did not have any allergic symptoms (NO), while 47.6% (n = 2188) reported RC, and 16.2% (n = 743), WZ. The proportion of cancer among NO groups was 5.43% (91/1669), among RC group, 7.63% (167/2188), and among WZ group, 11.23% (83/743) (RC group- OR 1.44 with 95% CI 1.00–2.08; p = 0.05 while for WZ group- OR 2.20 with 95%CI 1.27–3.80; p = 0.01). After adjusting for all the possible confounding variables including age, smoking, or COPD, having symptoms of RC (AOR 1.49 with 95%CI 1.12–2.36; p = 0.01) or WC (AOR 2.08 with 95%CI 1.11–3.89; p = 0.02) demonstrated consistent strong association with cancer. Among nonsmokers (n = 2505, 54.5%) only symptoms of RC showed association with cancer (AOR 1.51 with 95%CI 1.00–2.28; p = 0.05). Among former or current smokers (n = 2094, 45.5%), only symptoms of WZ demonstrated association with cancer (AOR 2.38 with 95%CI 1.16–4.87; p = 0.02). Among different types of cancers, odds of having breast cancer among participants with symptoms of RC or WZ were approximately twice the odds of having breast cancer among participants without any of these symptoms. AOR for RC group was 1.89 with 95%CI 1.04–3.42 and p = 0.04 while AOR for WC group was 2.08 with 95%CI 0.90–4.78 and p = 0.08.

**Conclusions:**

In summary, we found associations between common allergic symptoms like rhinitis/conjunctivitis and wheezing and prevalence of cancer, specifically between rhinitis/conjunctivitis and breast cancer that were not found in previous studies. Larger prospective studies are required to validate our findings.

## Introduction

Numerous epidemiological studies have investigated the association between allergic symptoms and cancer occurrence. There are two plausible theories that explain the relationship between allergy and cancer [1], [2]. One regards allergy as hyperstimulation of immune response to our body. Thus, allergy has potential to confer protective effect to cancer development with improved cancer cell immune surveillance. The suggested protective role of atopic allergy for lung and brain tumors supports this idea [3]–[5]. The other deems allergy as a constant state of inflammation in our body. Therefore, allergy may play a role in carcinogenesis via various inflammation pathway mediators. The fact that asthma has long been reported to be associated with increased lung cancer risk corroborates this notion [6]. Whether allergy actually moderates risk of cancer is still an area of controversy. Some epidemiologic studies have found positive association between allergy and cancer [6]–[12], while other studies have reported inverse association [3]–[5], [12]–[37]. Some studies showed no correlation between the two [1], [12], [38]–[45].

Many previous epidemiologic studies harbor limitations such as usage of different definition of allergy, small sample size of study population, and lack of control of possible confounders. Noticeably, most of previous reports have focused on symptoms of urticaria, bee sting allergy, hay fever, asthma, or atopic dermatitis/eczema. Few studies have examined the effect of common allergic symptoms such as conjunctivitis and rhinitis on cancer. In this study, we were able to obtain information on the symptoms of rhinitis and conjunctivitis as well as wheezing. In addition, we were able to control for various validated confounders such as smoking, asthma and chronic obstructive pulmonary disease (COPD) to better assess the effect of those symptoms on cancer in a NHANES III study participants, a reasonably large size cohort. We confined our analyses to female participants, first to better assess the effect of common allergic symptoms in a nonsmoker predominant female population, and, second to better evaluate the association between allergy and female hormone related tumors such as breast and uterine cancer that has previously been reported to be inconsistent and equivocal [12], [34], [45], [46].

## Methods

We did not obtain ethics approval for our study. Ethics approval was not necessary for this retrospective analysis of data available in literature in the already published NHANES III study, with anonymous subjects. For the same reason informed consent from participants involved in study could not be obtained and was not necessary being provided at the initial NHANES study.

### Study population

The Third National Health and Nutrition Examination Survey (NHANES III) is a stratified probability sample of the U.S. noninstitutionalized civilian population, conducted by the National Center for Health Statistics from 1988 through 1994. 4600 adults who were not pregnant and without any missing variables were selected out of 5,076 female adults aged ≥40 years who participated in both interview and physical examination in the NHANES III study. The prevalence of cancer excluding skin cancer was obtained by personal medical interview. Self-reported allergic symptoms were categorized into three categories: no symptoms (NO), rhinoconjunctivitis without wheezing (RC), and wheezing (WZ). First, the RC group was defined by those that answered “yes” to “During the past 12 months, have you had any episodes of stuffy, itchy or runny nose, or watery or itchy eyes?” questionnaire excluding those who had wheezing. Since allergic rhinoconjunctivitis can coexist with asthma or any nonspecific bronchial hyperreactivity, this question was designed to examine the effect of allergic rhinoconjunctivitis without bronchial overreactivity [40], [47]. Second, the WZ group was defined by those that answered yes to “Have you had any wheezing or whistling in your chest at any time in the past 12 months?” questionnaire to see the effect of nonspecific bronchial hypersensitivity irrespective of asthma. Asthma and COPD were controlled for in our multivariate analyses because these symptoms can be manifestations of such underlying disease. Other risk factors such as obesity, physical inactivity, and C-reactive protein level were defined by NHANES III questionnaire, results of physical examination and blood test.

Cancer was defined by self-reported physician diagnosed cancer as well as a cancer diagnosis listed on the records of hospital or nursing home, or on death certificate if hospital record of cancer was not available. Nonmelanoma skin cancers and diagnosis listed as questionable were excluded as cancer diagnosis.

### Statistical Analyses

All analysis was done with consideration of sampling methods and weights using STATA version 10 (College Station, TX). Pearson chi-square was used for categorical variables. Linear regression with Wald test was performed for continuous variables. Univariate and multivariate logistic regression was used to obtain odds ratios of cancer according to the existence of allergic symptoms. Stratified analyses were performed among smokers and nonsmokers. Interaction analyses were performed using Wald test. For multivariate analyses, adjustments were made for other possible confounding variables such as age, race, education, income, asthma, COPD, C-reactive protein, obesity, smoking, alcohol drinking, physical inactivity, and menopausal status. P value of 0.05 was used to determine statistical significance. Data used in the analyses are derived from the population estimates.

## Results

Among 4600 study participants, cancer was present in 7.41% (n = 341). The most common cancer was breast cancer (2.80%, n = 129), followed by uterine cancer (0.91%, n = 42), cervix cancer (0.89%, n = 41), and colon cancer (0.81%, n = 37). Interestingly, more than half of participants reported having common allergic symptoms including allergic rhinitis and conjunctivitis. Of the study group, 36.3% (n = 1669) did not have any allergic symptoms (NO), while 47.6% (n = 2188) reported RC, and 16.2% (n = 743), WZ. Demographic and clinical variables have been compared among people with the mentioned three different symptom categories (Table 1). Both RC and WZ groups were associated with having cancer and WZ group seemed to have stronger association with cancer than RC group (Table 2). The proportion of cancer among NO groups was 5.43% (91/1669), among RC group, 7.63% (167/2188), and among WZ group, 11.23% (83/743). Compared to the NO group, the odds ratio of cancer in the RC group was OR = 1.44 with 95% CI 1.00–2.08 and p = 0.05. For WZ group OR was 2.20 with 95% CI 1.27–3.80 and p = 0.01.

**Table 1:** Characteristics of study participants by allergic symptoms.

Characteristics	NO (n = 1669)	RC (n = 2188)	WZ (n = 743)	p value
**% of the population**	36.3	47.6	16.2	
**Age** (mean±SE, yrs)	59.5±0.69	57.6±0.55	57.4±0.74	0.02
**Race**				<0.01
White (%)	75.9	84.8	81.6	
African American (%)	11.5	7.9	9.0	
Hispanic (%)	4.3	2.3	3.4	
Etc. (%)	8.3	5.0	6.0	
**Education**				<0.01
≥12 years (%)	69.3	74.8	66.3	
9–11 years (%)	13.7	12.7	17.0	
<9 years (%)	17.2	12.5	16.7	
**Income**				0.01
>$ 25000 (%)	47.7	52.4	44.1	
$15000∼25000 (%)	19.3	19.9	18.9	
<$15000 (%)	24.5	19.6	30.0	
unknown (%)	8.5	8.1	7.0	
**BMI** (kg/m2)				<0.01
<18.5 (%)	3.0	2.5	2.1	
> = 18.5 & <25 (%)	38.2	42.5	27.9	
> = 25 & <30 (%)	30.6	30.8	31.1	
> = 30 (%)	28.2	24.2	38.9	
**Alcohol** (drinks)				0.13
never (%)	23.8	23.4	18.6	
mild (not current or < = 10/mo., %)	61.4	60.0	67.1	
heavy (>10/mo., %)	14.8	16.6	14.3	
**Smoking**				<0.01
never (%)	56.6	58.1	39.0	
former (%)	25.6	26.8	26.0	
active (%)	17.8	15.1	35.0	
**Physical activity**				<0.01
active (%)	76.6	82.3	70.2	
inactive (%)	23.4	17.7	29.8	
**Postmenopause** (%)	72.7	67.4	74.9	0.01
**CRP** (mean±SE, mg/dl)	0.47±0.02	0.48±0.02	0.67±0.04	0.01
**Asthma** (%)	3.4	5.4	29.0	<0.01
**COPD** (%)	4.3	8.3	33.7	<0.01
**Cancer** (%)	5.4	7.6	11.2	0.01

NO: no allergic symptoms; RC: symptoms of allergic rhinitis or conjunctivitis without wheezing; WZ: wheezing, SE: standard error.; BMI: body mass index; CRP: C reactive protein.

**Table 2:** Univariate and multivariate analyses of cancer risk factors among female study participants.

Clinical variables	univariate model			multivariate model		
	OR	95% CI	p value	AOR	95% CI	p value
**Age** (yrs)	1.02	1.01–1.04	<0.01	1.01	0.98–1.02	0.68
**Race**						
White	1			1		
African American	0.55	0.39–0.78	<0.01	0.65	0.44–0.98	0.04
Hispanic	0.44	0.25–0.75	<0.01	0.58	0.30–1.10	0.09
**Education**						
≥12 years	1			1		
9–11 years	1.15	0.73–1.82	0.54	1.02	0.62–1.70	0.93
<9 years	1.15	0.79–1.67	0.46	1.09	0.71–1.67	0.69
**Income**						
>$ 25000	1			1		
$15000∼25000	0.93	0.56–1.54	0.77	0.72	0.43–1.20	0.20
<$15000	1.19	0.85–1.68	0.30	0.85	0.58–1.24	0.38
**BMI** (kg/m2)						
BMI<18.5	1.79	0.66–.86	0.25	1.16	0.56–4.61	0.37
BMI> = 18.5 & <25	1			1		
BMI> = 25 & <30	0.84	0.57–1.22	0.35	0.73	0.51–1.05	0.09
BMI> = 30	0.82	0.51–1.32	0.41	0.74	0.45–1.21	0.23
**Alcohol**						
never	1			1		
mild (not current, or < = 10/mo.)	1.56	1.09–2.21	0.02	1.53	1.06–2.23	0.03
heavy (>10/mo.)	1.12	0.68–1.83	0.64	1.03	0.60–1.78	0.91
**Smoking**						
never	1			1		
former	1.62	1.11–2.36	0.01	1.37	0.94–2.00	0.10
active	1.24	0.79–1.96	0.34	0.95	0.56–1.60	0.85
**Physical inactivity**						
inactive	1.29	0.90–1.86	0.16	1.21	0.79–1.85	0.37
**Postmenopause**	7.52	3.20–17.67	<0.01	7.42	2.96–18.61	<0.01
**CRP** (mg/dl)	0.97	0.78–.20	0.78	0.87	0.68–1.10	0.23
**Asthma**	1.20	0.77–1.86	0.41	0.91	0.52–1.58	0.73
**COPD**	1.87	1.29–2.76	<0.01	1.24	0.78–1.96	0.36
NO	1			1		
RC	1.44	1.00–2.08	0.05	1.49	1.12–2.36	0.01
WZ	2.20	1.27–3.80	0.01	2.08	1.11–3.89	0.02

NO: no allergic symptoms; RC: symptoms of allergic rhinitis or conjunctivitis without wheezing; WZ: wheezing, SE: standard error.; BMI: body mass index; CRP: C reactive protein, OR: odds ratio; CI: confidence interval; AOR: adjusted odds ratio.

Amongst clinicopathological variables examined, race, alcohol, smoking, COPD, postmenopausal status, and WZ displayed statistically significant association with cancer (Table 2). Education, income, asthma, C-reactive protein level, obesity and physical inactivity were not found to be statistically significantly associated with cancer. RC exhibited borderline statistical significance in its association with cancer (p = 0.05). After adjusting for possible confounding variables such as race, alcohol, postmenopausal status, symptoms of rhinitis/conjunctivitis without wheezing and symptoms of wheezing remained statistically significant in their association with cancer (Table 2). Noticeably, besides postmenopausal status (AOR 7.42 with 95% CI 2.96–18.61; p<0.01), having symptoms of RC (AOR 1.49 with 95% CI 1.12–2.36; p = 0.01) or WC (AOR 2.08 with 95% CI 1.11–3.89; p = 0.02) demonstrated consistent strong association with cancer.

Interestingly, among nonsmokers (n = 2505, 54.5%), only symptoms of RC showed association with cancer (AOR 1.51 with 95% CI 1.00–2.28; p = 0.05), while, among former or current smokers (n = 2094, 45.5%), only symptoms of WZ demonstrated association with cancer (AOR 2.38 with 95% CI 1.16–4.87, p = 0.02) (Table 3). With subgroup analyses among different types of cancer, the association with symptoms of RC remained statistically significant with breast cancer which was also the most common cancer (Table 4). Odds of having breast cancer among participants with symptoms of RC or WZ were approximately twice the odds of having cancer without any symptoms (RC group- AOR 1.89 with 95% CI 1.04–3.42; p = 0.04 and WC group- AOR 2.08 with 95% CI 0.90–4.78; p = 0.08).

**Table 3:** Association between allergic symptoms and cancer among smokers and nonsmokers.

Smoking status	Allergic symptoms	Univariate model			Multivariate model		
		OR	95% CI	p value	AOR	95% CI	p value
Never (n = 2505)	NO	1			1		
	RC	1.33	0.90–2.00	0.15	1.51	1.00–2.28	0.05
	WZ	1.65	0.80–3.42	0.17	1.45	0.54–3.92	0.45
Former or Active (n = 2094)	NO	1			1		
	RC	1.57	0.88–2.79	0.12	1.49	0.82–2.68	0.18
	WZ	2.43	1.24–4.75	0.01	2.38	1.16–4.87	0.02

NO: no allergic symptoms; RC: symptoms of allergic rhinitis or conjunctivitis without wheezing; WZ: wheezing, OR: odds ratio; CI: confidence interval; AOR: adjusted odds ratio.

**Table 4:** Association between cancer subtypes and allergic symptoms among female study participants.

			Allergic Symptoms					
			RC			WZ		
Cancer types	numbers	proportion (%)	AOR	95% CI	p value	AOR	95% CI	p value
All	341	6.55	1.49	1.12–2.36	0.01	2.08	1.11–3.89	0.02
Breast	129	2.8	1.89	1.04–3.42	0.04	2.08	0.90–4.48	0.08
Uterine	42	0.91	1.01	0.43–2.40	0.97	1.94	0.44–8.57	0.37
Cervix	41	0.89	0.69	0.27–1.77	0.43	0.52	0.11–2.37	0.39
Colorectal	37	0.81	1.24	0.49–3.15	0.65	1.92	0.50–7.42	0.34

NO: no allergic symptoms; RC: symptoms of allergic rhinitis or conjunctivitis without wheezing; WZ: wheezing, OR: odds ratio; CI: confidence interval; AOR: adjusted odds ratio.

## Discussion

We have found that common allergic symptoms of rhinoconjunctivitis and wheezing were associated with cancer. Our NHANES III study results are consistent with previous studies that insinuated allergy as a risk factor for cancer [6]–[12]. Among them is a NHANES I follow up study from 1988, the first prospective study that controlled for the confounding factors such as age, gender, race, and smoking. It also illustrated a positive association between allergic symptoms and cancer (OR 1.40 with 95% CI 1.10–1.77). Interestingly, the association was stronger between hives and lymphatic-hematopoietic malignancies (OR 7.89 with 95% CI 3.13–19.89) [9]. While our study focused on self-reported symptoms of common allergic symptoms other than skin manifestations of allergy, it used prior physician made diagnosis of asthma, hay fever, drug allergy, and hives.

Interestingly, self-reported symptoms of rhinitis and conjunctivitis in the recent 12 months remained strongly associated with cancer even after adjusting for smoking and previous diagnosis of asthma or COPD, while neither asthma nor COPD showed any statistically significant association with cancer in a multivariate model. In addition, symptoms of wheezing illustrated stronger association with cancer than symptoms of rhinoconjunctivitis without wheezing, irrespective of smoking, asthma, and COPD. This finding may indicate that current or recent common symptoms of allergy may portray a stronger association with cancer than any past history of asthma, or COPD.

The strength of our study is the representative nature of our population based study cohort, which is derived from a national probability sample. This may have reduced probable selection bias. We also were able to control for possible confounders. Low socioeconomic status has been positively associated with both cancer [48] and allergy [49], [50]. People with higher education or higher income may engage in healthier lifestyle that protects themselves from possible occupational or environmental hazards linked to both allergy and cancer. Also, people with symptoms of allergy may be exhibiting different lifestyle patterns compared with people with no symptoms. Thus, smoking status, physical inactivity, alcohol consumption as well as obesity were controlled for.

Symptoms of wheezing may result not only from allergy but from other causes including COPD, postnasal drip syndrome, pneumonia, vocal cord dysfunction, congestive heart failure, and exposure to irritants. Although asthma is one of the most common causes for wheezing, a finding of wheezing is neither sensitive nor specific for asthma [51]. In that regards, symptoms of RC may be a better index for allergy than WZ. In addition, people with very mild or well controlled asthma may not have experienced any symptoms of WZ in the past 12 months. Also, postnasal drip syndrome from allergic rhinitis may cause WZ. Thus, in our study, we focused more on the symptom of WZ, rather than prior diagnosis of asthma. Therefore, it is interesting to note that symptoms of RC, not WZ, are linked with cancer among nonsmokers. Accordingly, in our multivariate model, we controlled for asthma and COPD when assessing the association between WZ and cancer.

Smoking is an important factor to take into consideration in assessing allergic symptoms. In people with smoking history, greater misclassification of asthma may occur. Possibly, people with allergic symptoms may opt not to smoke, or may quit smoking more frequently than people with no such symptoms [52]. Therefore, we did stratified analyses according to smoking status. Since we focused on female participants only, there were more never smokers (54.5%) than former or current smokers (45.5%) (Table 4). Intriguingly, among nonsmokers, the association between symptoms of RC and cancer remained statistically significant when controlled for possible confounders, indicating that mild common allergic symptoms may be linked with cancer more so in nonsmokers. To the contrary, among smokers, only symptoms of WZ were associated with cancer while symptoms of RC were not. This may mean that smoking may have affected the risk for developing symptoms of WZ or cancer in a differential fashion. However, there was no significant interaction of smoking on the association between allergy and cancer (Wald test p = 0.78)

Noticeably, breast cancer seems to be associated with symptoms of RC, rather than WZ. Consistent with our study, two prospective cohort studies including NHANES I follow up study found the same positive association between allergy and breast cancer [9], [46]. Breast cancer has been initially suggested to have inverse association with asthma, but most recent studies have reported no significant association [12], [34], [45], [46] with common allergic symptoms. While allergy was linked with reduced breast cancer risk for people diagnosed with breast cancer between age of 35 and 45 [21], average age for our study participants was close to 60 (Table 1).

The role of allergy in carcinogenesis is still unclear. There are, however, multiple plausible mechanisms to explain our findings. First, contribution of chronic inflammation to carcinogenesis and tumor progression has been well established [53]. Allergy is a chronic condition that involves release of pro-inflammatory cytokines and presence of asthma has been related to the risk of developing lung cancer [6]. Interestingly, CRP, a known marker for inflammation, was not found to be associated with cancer in our study (Table 2). However, the role of immune response from allergy may be more complex depending on what type of immune cells are primarily involved in the reaction and where the carcinogenesis occurs [1], [2]. Second, rhinitis or asthma may cause reduced clearance of carcinogens that are found in the airway. Mucociliary dysfunction has been described in asthmatics [54]. Third, estrogen may play a role. Allergy after puberty is more common and more severe among females and estrogen is known to augment humoral immune reaction and activate mast cells [55], [56]. It is noteworthy that breast cancer was associated with having common symptoms of allergy and that no significant association between allergy and cancer could be found among NHANES III male participants (data not shown).

We identified the following limitations in our study. First, our study is a cross sectional study enabling a spurious positive association to result if undiagnosed cancer was causing any symptoms of RC or WZ. Although undiagnosed lung tumor may cause wheezing, the chance of having undiagnosed cancer in the lung causing wheezing is deemed low. In addition, symptoms of RC are not typical symptoms of undiagnosed cancer. Second, ascertainment bias may be present since data are built mostly from answers from questionnaires, or self-reported symptoms. Presence of allergy, asthma or COPD are self reported by the participants and the diagnosis or severity of the symptoms have not been confirmed by any objective measures such as serum IgE levels or pulmonary function test. Although having symptoms of RC or WZ in the last 12 months may not necessarily mean that allergy is present, validity of self-reported allergic symptoms has been reported previously [57]. Third, differential recall bias may be present in assessing allergic symptoms between patients with cancer and without cancer. Fourth, there could be other missed confounders that can skew our results. For instance, antihistamines used in allergy have been suggested to be linked with cancer [58], although inconclusive [59]. Also use of inhaled corticosteroid has been associated with reduction of lung cancer risk in COPD patients [60].

In summary, we found statistically significant associations between common allergic symptoms like rhinitis/conjunctivitis and wheezing and all cancers, specifically between rhinitis/conjunctivitis and breast cancer not found in previous studies. Larger prospective studies are required to validate our findings.

## References

[1] Lindelof B, Granath F, Tengvall-Linder M, Ekbom A (2005) Allergy and cancer. Allergy 60 (9) 1116–20. 16076294 10.1111/j.1398-9995.2005.00808.x

[2] Turner MC, Chen Y, Krewski D, Ghadirian P (2006) An overview of the association between allergy and cancer. Int J Cancer 118 (12) 3124–3132. 16395696 10.1002/ijc.21752

[3] Brenner AV, Linet MS, Fine HA, Shapiro WR, Selker RG, et al (2002) History of allergies and autoimmune diseases and risk of brain tumors in adults. Int J Cancer 99 (2) 252–259. 11979441 10.1002/ijc.10320

[4] Castaing M, Youngson J, Zaridze D, Szeszenia-Dabrowska N, Rudnai P, et al (2005) Is the risk of lung cancer reduced among eczema patients? Am J Epidemiol 162 (6) 542–547. 16093291 10.1093/aje/kwi241

[5] Wigertz A, Lonn S, Schwartzbaum J, Hall P, Auvinen A, et al (2007) Allergic conditions and brain tumor risk. Am J Epidemiol 166 (8) 941–950. 17646205 10.1093/aje/kwm203

[6] Santillan AA, Camargo CA Jr, Colditz GA (2003) A meta-analysis of asthma and risk of lung cancer (United States). Cancer Causes Control 14 (4) 327–334. 12846363 10.1023/a:1023982402137

[7] Gallagher RP, Spinelli JJ, Elwood JM, Skippen DH (1983) Allergies and agricultural exposure as risk factors for multiple myeloma. Br J Cancer 48 (6) 853–857. 6652026 10.1038/bjc.1983.277 PMC2011571

[8] Logan J, Saker D (1953) The incidence of allergic disorders in cancer. N Z Med J 52 (289) 210–212. 13087692

[9] McWhorter WP (1988) Allergy and risk of cancer. A prospective study using NHANES I followup data. Cancer 62 (2) 451–455. 3383143 10.1002/1097-0142(19880715)62:2<451::aid-cncr2820620234>3.0.co;2-d

[10] Robinette CD, Fraumeni JF Jr (1978) Asthma and subsequent mortality in World War II veterans. J Chronic Dis 31 (9–10) 619–624. 744792 10.1016/0021-9681(78)90022-x

[11] Sigurgeirsson B, Lindelof B (1991) Positive patch test and cancer: an epidemiological study of 5858 patients. Am J Contact Dermatitis 2: 235–238.

[12] Vesterinen E, Pukkala E, Timonen T, Aromaa A (1993) Cancer incidence among 78,000 asthmatic patients. Int J Epidemiol 22 (6) 976–982. 8144310 10.1093/ije/22.6.976

[13] Alderson M (1974) Mortality from malignant disease in patients with asthma. Lancet 2 (7895) 1475–1477. 4140394 10.1016/s0140-6736(74)90217-7

[14] Allegra J, Lipton A, Harvey H, Luderer J, Brenner D, et al (1976) Decreased prevalence of immediate hypersensitivity (atopy) in a cancer population. Cancer Res 36 (9 pt.1) 3225–3226. 975086

[15] Amlot PL, Slaney J, Brown R (1983) Atopy–a favourable prognostic factor for survival in Hodgkin's disease. Br J Cancer 48 (2) 209–215. 6882661 10.1038/bjc.1983.176 PMC2011439

[16] Bilek O, Munzarova M, Zahalkova M (1975) Atopy and cancer. Neoplasma 1 22 (4) 441–444. 1196427

[17] Cockcroft DW, Klein GJ, Donevan RE, Copland GM (1979) Is there a negative correlation between malignancy and respiratory atopy? Ann Allergy 43 (6) 345–347. 517816

[18] Fisherman EW (1960) Does the allergic diathesis influence malignancy? J Allergy 31: 74–78. 13823262 10.1016/0021-8707(60)90026-5

[19] Ford RM (1978) Primary lung cancer and asthma. Ann Allergy 40 (4) 240–242. 637382

[20] Gabriel R, Dudley BM, Alexander WD (1972) Lung cancer and allergy. Br J Clin Pract 26 (5) 202–204. 4114639

[21] Hedderson MM, Malone KE, Daling JR, White E (2003) Allergy and risk of breast cancer among young women (United States). Cancer Causes Control 14 (7) 619–626. 14575359 10.1023/a:1025686913626

[22] Holly EA, Eberle CA, Bracci PM (2003) Prior history of allergies and pancreatic cancer in the San Francisco Bay area. Am J Epidemiol 158 (5) 432–441. 12936898 10.1093/aje/kwg174

[23] Johnson KJ (1966) The relation of cancer to allergy. J Lancet 86 (1) 5–11. 5900921

[24] Kallen B, Gunnarskog J, Conradson TB (1993) Cancer risk in asthmatic subjects selected from hospital discharge registry. Eur Respir J 6 (5) 694–697. 8519380

[25] Mackay WD (1966) The incidence of allergic disorders and cancer. Br J Cancer 20 (3) 434–437. 5922249 10.1038/bjc.1966.52 PMC2008019

[26] McDuffie HH (1991) Atopy and primary lung cancer. Histology and sex distribution. Chest 99 (2) 404–407. 1989803 10.1378/chest.99.2.404

[27] McDuffie HH, Cockcroft DW, Talebi Z, Klaassen DJ, Dosman JA (1988) Lower prevalence of positive atopic skin tests in lung cancer patients. Chest 93 (2) 241–246. 3338290 10.1378/chest.93.2.241

[28] Meers PD (1973) Allergy and cancer. Lancet 1 (7808) 884–885. 10.1016/s0140-6736(73)91452-9 4123430

[29] Sanchez-Borges M, de Orozco A, Arellano S, de Gallego V, Avila-Millian E, et al (1986) Preventive role of atopy in lung cancer. Clin Immunol Immunopathol 41 (3) 314–319. 3780049 10.1016/0090-1229(86)90002-4

[30] Schuz J, Kaletsch U, Meinert R, Kaatsch P, Michaelis J (1999) Association of childhood leukaemia with factors related to the immune system. Br J Cancer 80 (3–4) 585–590. 10408870 10.1038/sj.bjc.6690395 PMC2362320

[31] Schuz J, Morgan G, Bohler E, Kaatsch P, Michaelis J (2003) Atopic disease and childhood acute lymphoblastic leukemia. Int J Cancer 105 (2) 255–260. 12673688 10.1002/ijc.11054

[32] Severson RK, Davis S, Thomas DB, Stevens RG, Heuser L, et al (1989) Acute myelocytic leukemia and prior allergies. J Clin Epidemiol 42 (10) 995–1001. 2809658 10.1016/0895-4356(89)90165-0

[33] Shapiro S, Fedullo A (1973) Allergy and cancer. The Lancet 301 (7811) 1055–1056. 4122127

[34] Turner MC, Chen Y, Krewski D, Ghadirian P, Thun MJ, et al (2005) Cancer mortality among US men and women with asthma and hay fever. Am J Epidemiol 162 (3) 212–221. 15987724 10.1093/aje/kwi193

[35] Ure DM (1969) Negative assoication between allergy and cancer. Scott Med J 14 (2) 51–54. 5812963 10.1177/003693306901400203

[36] Vena JE, Bona JR, Byers TE, Middleton E Jr, Swanson MK, et al (1985) Allergy-related diseases and cancer: an inverse association. Am J Epidemiol 122 (1) 66–74. 4014202 10.1093/oxfordjournals.aje.a114087

[37] Wen W, Shu XO, Linet MS, Neglia JP, Potter JD, et al (2000) Allergic disorders and the risk of childhood acute lymphoblastic leukemia (United States). Cancer Causes Control 11 (4) 303–307. 10843442 10.1023/a:1008958724739

[38] Eriksson NE, Holmen A, Hogstedt B, Mikoczy Z, Hagmar L (1995) A prospective study of cancer incidence in a cohort examined for allergy. Allergy 50 (9) 718–722. 8546265 10.1111/j.1398-9995.1995.tb01212.x

[39] Boffetta P, Ye W, Boman G, Nyren O (2002) Lung cancer risk in a population-based cohort of patients hospitalized for asthma in Sweden. Eur Respir J 19 (1) 127–133. 11843311 10.1183/09031936.02.00245802

[40] Bousquet J, Van Cauwenberge P, Khaltaev N (2001) Allergic rhinitis and its impact on asthma. J Allergy Clin Immunol 108 (5 Suppl) S147–334. 11707753 10.1067/mai.2001.118891

[41] Hughes WF, Raitz RL (1979) A comparison of cancer occurrence in allergic and nonallergic populations. Ann Allergy 43 (3) 163–164. 475066

[42] Ming ME, Levy R, Hoffstad O, Filip J, Abrams BB, et al (2004) The lack of a relationship between atopic dermatitis and nonmelanoma skin cancers. J Am Acad Dermatol 50 (3) 357–362. 14988675 10.1016/j.jaad.2003.09.024

[43] Petroianu A, Chaves DN, De Oliveira O Jr (1995) Comparative incidence of allergy in the presence or absence of cancer. J Int Med Res 23 (5) 358–363. 8529778 10.1177/030006059502300505

[44] Schwartzbaum J, Jonsson F, Ahlbom A, Preston-Martin S, Lönn S, et al (2003) Cohort studies of association between self-reported allergic conditions, immune-related diagnoses and glioma and meningioma risk. Int J Cancer 106 (3) 423–428. 12845684 10.1002/ijc.11230

[45] Talbot-Smith A, Fritschi L, Divitini ML, Mallon DF, Knuiman MW (2003) Allergy, atopy, and cancer: a prospective study of the 1981 Busselton cohort. Am J Epidemiol 157 (7) 606–612. 12672680 10.1093/aje/kwg020

[46] Mills PK, Beeson WL, Fraser GE, Phillips RL (1992) Allergy and cancer: organ site-specific results from the Adventist Health Study. Am J Epidemiol 136 (3) 287–295. 1415150 10.1093/oxfordjournals.aje.a116494

[47] Nathan RA (2007) The burden of allergic rhinitis. Allergy Asthma Proc 28 (1) 3–9. 17390749 10.2500/aap.2007.28.2934

[48] Boyd C, Zhang-Salomons JY, Groome PA, Mackillop WJ (1999) Associations between community income and cancer survival in Ontario, Canada, and the United States. J Clin Oncol 17 (7) 2244–2255. 10561282 10.1200/JCO.1999.17.7.2244

[49] Bergmann RL, Edenharter G, Bergmann KE, Lau S, Wahn U (2000) The Multicenter Allergy Study Research G (2000) Socioeconomic status is a risk factor for allergy in parents but not in their children. Clinical & Experimental Allergy 30 (12) 1740–1745. 11122212 10.1046/j.1365-2222.2000.00927.x

[50] Lewis SA, Weiss ST, Platts-Mills TA, Syring M, Gold DR (2001) Association of specific allergen sensitization with socioeconomic factors and allergic disease in a population of Boston women. J Allergy Clin Immunol 107 (4) 615–622. 11295648 10.1067/mai.2001.113523

[51] Pratter MR, Hingston DM, Irwin RS (1983) Diagnosis of bronchial asthma by clinical evaluation. An unreliable method. Chest 84 (1) 42–47. 6861547 10.1378/chest.84.1.42

[52] Mackillop WJ, Zhang-Salomons J, Boyd CJ, Groome PA (2000) Associations between community income and cancer incidence in Canada and the United States. Cancer 89 (4) 901–912. 10951356 10.1002/1097-0142(20000815)89:4<901::aid-cncr25>3.0.co;2-i

[53] Keibel A, Singh V, Sharma MC (2009) Inflammation, microenvironment, and the immune system in cancer progression. Curr Pharm Des 15 (17) 1949–1955. 19519435 10.2174/138161209788453167

[54] Wanner A, Salathe M, O'Riordan TG (1996) Mucociliary clearance in the airways. Am J Respir Crit Care Med 154 (6 Pt 1) 1868–1902. 8970383 10.1164/ajrccm.154.6.8970383

[55] Chen W, Mempel M, Schober W, Behrendt H, Ring J (2008) Gender difference, sex hormones, and immediate type hypersensitivity reactions. Allergy 63 (11) 1418–1427. 18925878 10.1111/j.1398-9995.2008.01880.x

[56] Jensen-Jarolim E, Untersmayr E (2008) Gender-medicine aspects in allergology. Allergy 63 (5) 610–615. 18394135 10.1111/j.1398-9995.2008.01645.x PMC2999751

[57] Toren K, Brisman J, Jarvholm B (1993) Asthma and asthma-like symptoms in adults assessed by questionnaires. A literature review. Chest 104 (2) 600–608. 7802735 10.1378/chest.104.2.600

[58] Brandes LJ, Warrington RC, Arron RJ, Bogdanovic RP, Fang W, et al (1994) Enhanced cancer growth in mice administered daily human-equivalent doses of some H1-antihistamines: predictive in vitro correlates. J Natl Cancer Inst 86 (10) 770–775. 7909571 10.1093/jnci/86.10.770

[59] Nadalin V, Cotterchio M, Kreiger N (2003) Antihistamine use and breast cancer risk. Int J Cancer 106 (4) 566–568. 12845653 10.1002/ijc.11240

[60] Parimon T, Chien JW, Bryson CL, McDonell MB, Udris EM, et al (2007) Inhaled corticosteroids and risk of lung cancer among patients with chronic obstructive pulmonary disease. Am J Respir Crit Care Med 175 (7) 712–719. 17185647 10.1164/rccm.200608-1125OC PMC1899285

